# Transition from In-Person to Online Boards—An Exploratory Pilot Study on Pituitary Tumor Board Meetings

**DOI:** 10.3390/jcm15083132

**Published:** 2026-04-20

**Authors:** Carina Obermüller, Zoran Erlic, Felix Beuschlein, Andrea Bink

**Affiliations:** 1 Department of Neuroradiology, Clinical Neuroscience Center, University Hospital Zurich, University of Zurich, 8091 Zurich, Switzerland; 2 Department of Endocrinology, Diabetology and Clinical Nutrition, University Hospital Zurich, University of Zurich, 8091 Zurich, Switzerland

**Keywords:** telemedicine, endocrine tumors, medical education, clinical decision-making, interdisciplinary communication, tumor

## Abstract

**Objectives**: The transition from in-person to online formats for clinical case discussions has transformed the way medical professionals collaborate and share knowledge. The current pilot study investigates the potential impact of this shift by comparing the experiences of specialists and residents who had practiced both online and in-person pituitary tumor boards compared to the experiences of those who only experienced the online format. **Methods**: A cohort of 15 participants, including 10 specialists and 5 residents, provided insights through structured surveys and free-text responses. **Results**: The results indicate differences in the perception of the duration and participation dynamics in online pituitary tumor boards between both groups, with online boards facilitating the discussion of more cases. Overall participant engagement was mostly perceived as the same; a subset reported reduced perceived engagement, particularly among senior professionals. **Conclusions**: These findings highlight how prior experience can affect the perceived effectiveness, level of engagement and duration of online formats, providing valuable hypothesis-generating input for optimizing future medical board meetings.

## 1. Introduction

Digital communication technologies have profoundly transformed the way medical professionals collaborate on complex cases and engage in interdisciplinary consultations [1]. Traditionally, case discussions, including specialized boards, were held in person, facilitating direct interaction and the in-depth exchange of expertise. Pituitary tumor boards focus specifically on cases involving pituitary disorders, bringing together endocrinologists, neurosurgeons, neuroradiologists, pathologists, and other specialists to collaboratively discuss diagnostic, therapeutic, and management strategies. As digital communication technologies have advanced, these boards can now be conducted completely virtually, promising greater accessibility and logistical ease. This shift, however, may bring both advantages and challenges.

The COVID-19 pandemic has catalyzed the widespread adoption of virtual formats for many medical interactions, both between patients and medical care providers and among medical care providers [2,3,4,5]. This also included pituitary tumor boards, as in-person gatherings were restricted to minimize the spread of the virus. Many institutions rapidly adopted online platforms, allowing interdisciplinary meetings to continue with minimal disruption. These virtual boards offer flexibility, enabling participation from a broader range of specialists across locations and allowing for reduced travel time and costs. As a result, the convenience and adaptability of virtual boards have led many institutions to continue utilizing them even beyond the height of the pandemic.

Despite these advantages, there have been indications that the shift from in-person to online meetings may impact the dynamics and quality of these critical discussions. For example, prior research suggested that virtual settings allowed for quicker expert analysis of cases compared to traditional in-person meetings [6]. This shift may be attributed to logistical improvements; however, the remote format may also impact factors such as the depth of the case analysis, the duration of the meetings, and the level of participant engagement. The nuances of non-verbal communication, spontaneous discussion, and collaborative problem-solving that characterize in-person meetings may be challenging to replicate in an online format.

These challenges in replicating the rich, interactive nature of in-person meetings are echoed in broader media psychology research, which provides a framework for understanding the complexities of virtual collaboration. The adoption of any new communication technology is shaped not only by its objective features but also by users’ ‘anticipated affordances’—the speculative judgments and fears about how a technology might be used or misused, which can influence adoption before any rational assessment of its utility [7]. Once in use, the effectiveness of a communication medium is influenced by a complex web of user goals that extend beyond simple task efficiency to include the maintenance of relational and community bonds, which are crucial in collaborative professional settings [8]. Furthermore, sustained and effective participation in these new digital environments can be conceptualized as a learned skill, requiring users to self-regulate their engagement over time in a cyclical process of planning, performance, and reflection [9]. In more immersive settings, key predictors for successful engagement include not only the system’s usability but also the degree to which it fosters a psychological sense of presence, immersion, and user agency—the ability to interact meaningfully within the virtual space [10]. 

The current study represents the initial work in a broader analysis of online boards, specifically investigating how prior experience—or the lack thereof—influences the perception of digital collaboration. We hypothesize that the anticipated affordances of online boards and the degree to which participants successfully employ self-regulated learning to adapt to these formats will differ significantly between those who have an in-person benchmark (IG) and those who have only experienced the virtual environment (OG).

## 2. Materials and Methods

### 2.1. Participant Selection

The pituitary tumor board at our institution was chosen for this study as this board was performed immediately online after the first cases of COVID-19 in Europe and is the only board in our institution that is performed exclusively online to this day.

Formal ethics approval was not necessary according to the Kantonale Ethikkommission Zürich (KEK), as the study was an anonymous quality evaluation and satisfaction survey involving healthcare professionals, which does not fall under the scope of the Human Research Act. Human Ethics and Consent to Participate declarations are not applicable. By filling in the questionnaire, each participant gave consent for their data to be used in an anonymized way. The pituitary tumor board evaluated in the current study took place once a month, and was transformed into a fully online format at the beginning of 2020. The actual meeting lasted between 60 and 120 min, with 15 to 30 patient cases discussed per meeting, with an increase in cases from an average of 10 cases in 2019 to an average of 20 cases in 2024. Attendance per meeting was between 15 and 20 participants, whereby 8 to 10 participants constituted senior doctors and the remaining participants were junior doctors. Perceived adherence to recommendations was followed, whereas clinical outcome was not addressed as a topic in this study. The survey was sent out in July 2024 to 53 potential participants who attended or expressed interest in attending the pituitary tumor board at the University Hospital of Zurich; a total of 15 of those potential participants (28%) participated in this study. This is in line with response rates for physician surveys found in the literature [11]. All participants were affiliated with the University Hospital of Zurich or its cooperating partners within the region and had varying degrees of experience with both the online-only and in-person-only formats of the pituitary tumor board.

### 2.2. Survey Design and Data Collection

The survey questions were conceptualized and reviewed by all authors. The survey was conceptualized through a structured internal review by all authors. No formal external validation, pilot testing, or reliability analysis was performed. Free-text responses were analyzed using a thematic coding approach with independent review by two authors to ensure consistency. After review, the survey was disseminated. The complete survey, with all possible answer options and the order of appearance of the questions, can be found in the Appendix A in Table A1, Table A2 and Table A3.

Participants were asked a series of quantitative and qualitative questions regarding their experiences with both formats. The primary questions aimed to assess differences in meeting duration, perceived participation dynamics between junior and senior staff, and the overall volume of patient cases discussed. Participants who had experienced both formats were asked to provide detailed comparisons, while those who had only participated in the online board were asked to focus on their online experiences.

Free-text responses allowed for additional qualitative insights into perceived differences between the two formats, particularly regarding discussion quality and case volume.

### 2.3. Statistics

Statistical analysis was performed in Python 3.12.1, Python Software Foundation, www.python.org. Data analysis included both descriptive and exploratory inferential statistics. Exact 95% confidence intervals (Clopper-Pearson) were calculated for key proportions. Subgroup comparisons between the IG and OG were performed using Fisher’s Exact Test (2 × 2) or Chi-square (matrices > 2 × 2) (*p* < 0.05 considered significant).

## 3. Results

### 3.1. Participant Demographics

The cohort consisted of 67% specialists (n = 10) and 33% residents (n = 5). Of the participants, 67% (n = 10) had experienced both the online and in-person formats (IG, online and in-person group) of the pituitary tumor board, while 33% (n = 5) had only experienced the online format (OG, online-only group). There were nine specialists in the IG and one in the OG.

**Figure 1:**
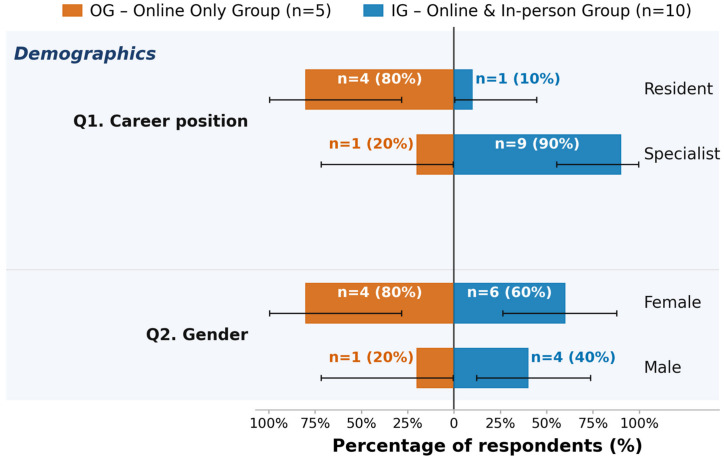
Participant demographics (career position Q1; gender Q2), stratified by group. OG = online-only group (n = 5, left side); IG = online and in-person group (n = 10, blue bars, right side). Bar lengths represent the within-group percentage; labels show absolute counts and percentages (n = X (XX%)). Error bars: exact 95% Clopper–Pearson confidence intervals. Zero-count options are omitted.

The participants’ experience in their current career position ranged from 2 to 25 years, with more years of experience being reported more frequently. The average years practicing was 9 years; the median was 8 years (interquartile range 2.0–13.5). Details of participant characteristics are shown in Table 1 and Figure 1.

### 3.2. Overall Quantitative Results

Regarding technical quality, 87% (n = 13) of respondents reported adequate access to digital case documentation, and 93% (n = 14) reported no technical issues. In terms of communication quality, 93% (n = 14) participants indicated privacy was maintained, while responses regarding the adequate conveyance of non-verbal cues included eight “Yes,” three “No,” and four “No opinion.” Participation data by demographics showed that all 15 respondents rated the perceived involvement of both male and female participants as “Same.” For participation by seniority, 80% (n = 12) rated perceived senior involvement as “Same” (20% “Less” (n = 3)), and 93% (n = 14) rated perceived junior involvement as “Same” (7% “Less” (n = 1)). In the domain of clinical outcomes, 53% (n = 8) respondents reported “No access to this information” regarding adherence to board decisions; 73% (n = 11) reported “No” to more elaborative or cautious follow-up decisions, and 87% (n = 13) reported “No” to more aggressive treatment decisions. Concerning efficiency and preferences, 80% (n = 12) respondents preferred the online format. The reported perceived duration of online tumor boards was most frequently “Less than 5 min difference” (53%, n = 8).

### 3.3. Subgroup Analysis Comparing OG and IG

#### 3.3.1. Technical and Communication Feasibility

A total of 60% (n = 3) of participants in the OG and 50% (n = 5) in the IG reported not having access to the information showing whether board decisions were adhered to. A total of 20% (n = 1) of participants in the OG and 10% (n = 1) in the IG reported that board decisions were adhered to more often following online boards, while the remaining participants reported adherence to be the same.

A total of 10% (n = 1) of participants in the IG group indicated not having adequate access to digital documentation and information on the case during online boards. In the OG, 80% (n = 4) reported having access, while the remaining 20% (n = 1) had no opinion.

**Figure 2:**
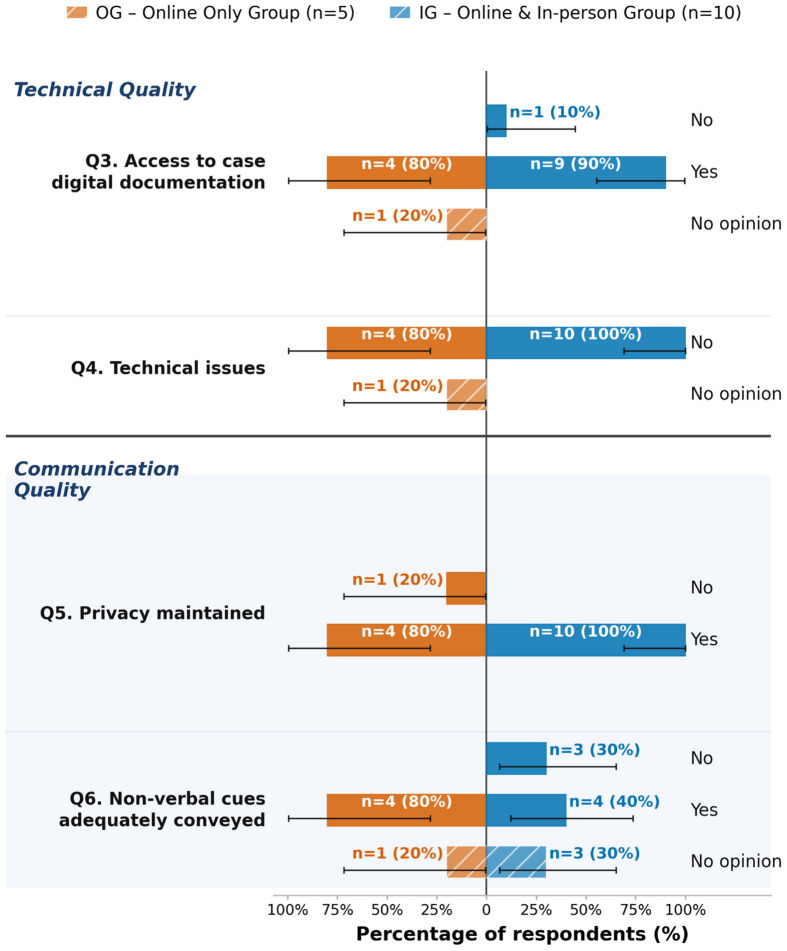
Exploratory analysis of technical and communication feasibility (Questions 3–6 (Q3–Q6)), stratified by group. OG = online-only group (n = 5, left side); IG = online and in-person group (n = 10, blue bars, right side). Bar lengths represent the within-group percentage; labels show absolute counts and percentages (n = X (XX%)). Zero-count options are omitted. Error bars: exact 95% Clopper–Pearson confidence intervals. All results are exploratory.

Both groups did not report technical issues being a hindrance to participation in online boards. A total of 20% (n = 1) of participants in the OG found privacy and confidentiality to be inadequately maintained in online boards, while this was not reported in the IG. A total of 30% (n = 3) of participants in the IG group reported that they found non-verbal cues to be insufficiently conveyed and interpreted in online boards, while none of the participants in the OG found this to be an issue. Details can be found in Figure 2. Exploratory testing using Fisher’s Exact or Chi-square tests did not yield significant results (see Table S1 in the Supplementary Material).

#### 3.3.2. Participation Dynamics

**Figure 3:**
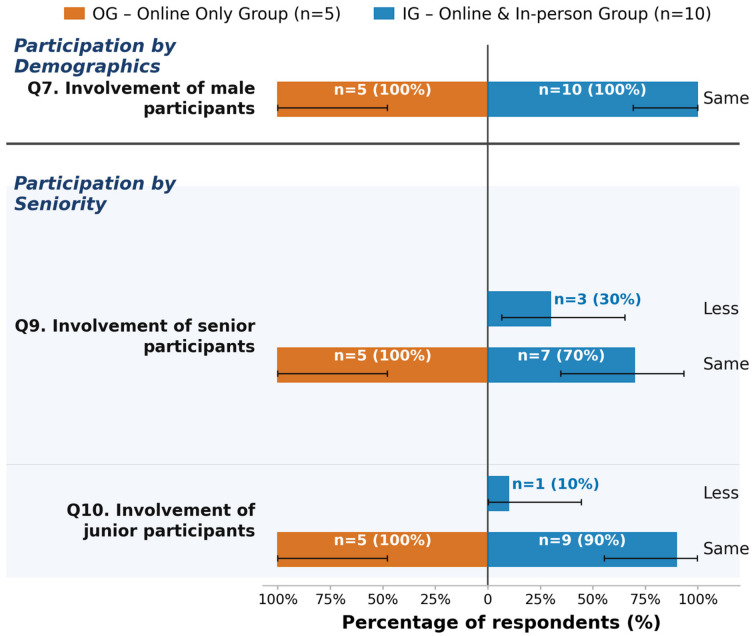
Exploratory analysis of participation dynamics (involvement of male/female/junior/senior participants, Question 7–Question 10 (Q7–Q10)) across online tumor board sessions, stratified by group. OG = online-only group (n = 5, left side); IG = online and in-person group (n = 10, blue bars, right side). Bar lengths represent the within-group percentage; labels show absolute counts and percentages (n = X (XX%)). Zero-count options are omitted. Error bars: exact 95% Clopper–Pearson confidence intervals. All results are exploratory.

Both groups reported the same level of perceived involvement in the decision and implementation process in online boards compared to in-person boards for both male and female participants. Although the majority reported the perceived involvement of senior and junior participants to be the same, 30% (n = 3) in the IG perceived senior participants to be less involved in the decision and implementation process in online boards compared to in-person boards. In the OG, no difference was reported. A total of 10% (n = 1) of participants in the IG perceived junior participants to be less involved in the decision and implementation process in online boards compared to in-person boards, while the remaining 90% (n = 9) reported no difference. Details can be found in Figure 3. Exploratory testing using Fisher’s Exact or Chi-square tests did not yield significant results (see Table S2 in the Supplementary Material).

#### 3.3.3. Clinical Outcomes and Efficiency

**Figure 4:**
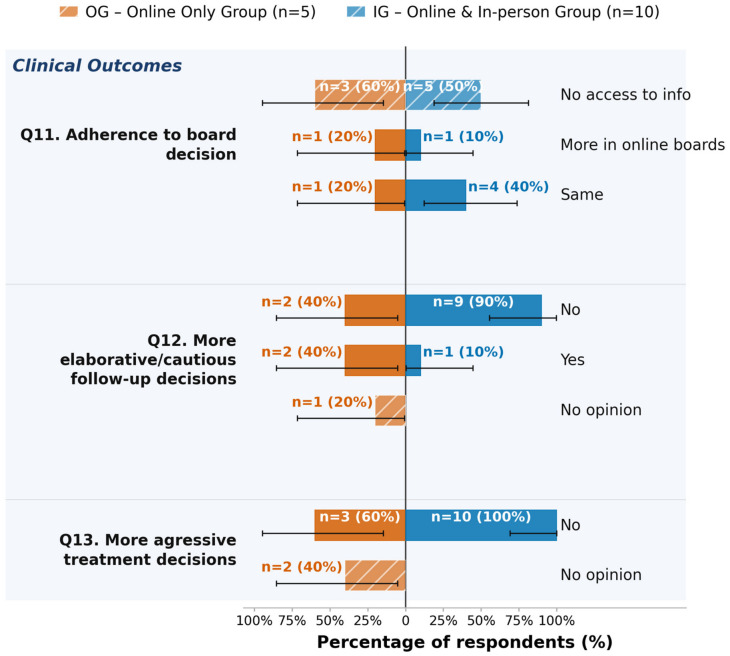
Exploratory analysis of perceived clinical outcomes (Question 11–Question 13 (Q11–Q13)), stratified by group. OG = online-only group (n = 5, left side); IG = online and in-person group (n = 10, blue bars, right side). Bar lengths represent the within-group percentage; labels show absolute counts and percentages (n = X (XX%)).

Concerning follow-up management, 10% (n = 1) of participants in the IG believed online boards led to a more cautious and elaborative follow-up, while 90% (n = 9) did not believe this was the case. In the OG, 40% (n = 2) of participants believed online boards led to a more cautious and elaborative follow-up, while 40% (n = 2) did not believe this was the case and 20% (n = 1) had no opinion on this question. A total of 100% (n = 10) of participants in the IG reported treatment decisions were not leaning towards a more aggressive approach in online boards compared to in-person boards. In the OG, only 60% (n = 3) of participants reported this, while the remaining 40% (n = 2) had no opinion on this question. Overall, the answer option “no opinion” was selected more frequently in the OG than in the IG. Details can be found in Figure 4. Exploratory testing using Fisher’s Exact or Chi-square tests did not yield significant results (see Table S3 in the Supplementary Material).

Zero-count options are omitted. All outcomes reflect participants’ subjective perception only. Error bars: exact 95% Clopper–Pearson CIs. All results are exploratory.

**Figure 5:**
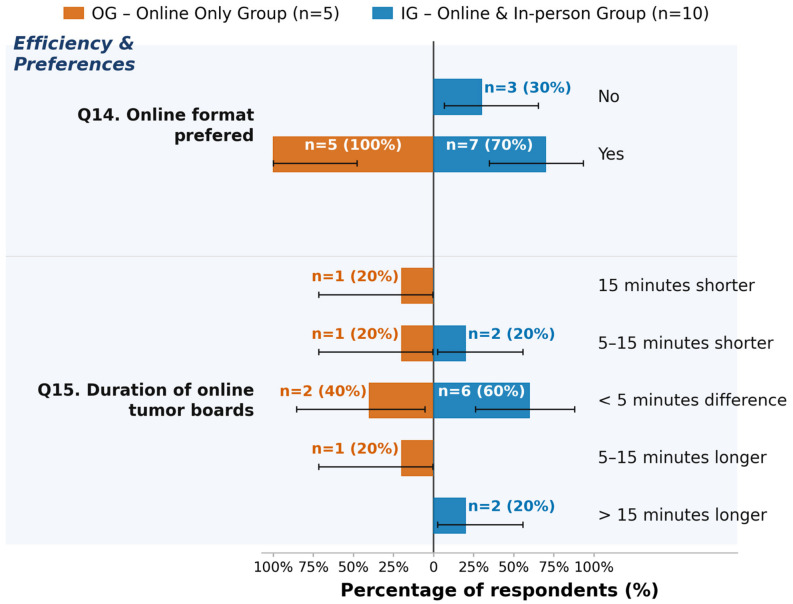
Exploratory analysis of efficiency and preferences (Question 14–Question 15 (Q14–Q15)), stratified by group. OG = online-only group (n = 5, left side); IG = online and in-person group (n = 10, blue bars, right side). Bar lengths represent the within-group percentage; labels show absolute counts and percentages (n = X (XX%)). All outcomes reflect participants’ subjective perception only. Error bars: exact 95% Clopper–Pearson CIs. All results are exploratory.

A total of 30% (n = 3) of participants in the IG group reported that they prefer in-person boards, while the remaining 70% (n = 7) reported preferring online boards. In the OG, 100% (n = 5) of participants reported preferring online boards. The perception of duration varied. A total of 40% (n = 2) of participants in the OG and 60% (n = 6) in the IG reported the perceived duration to be comparable in in-person and in online boards. In both groups, 20% (OG: n = 1, IG: n = 2) of participants perceived online boards to be 5 to 15 min shorter than in-person boards. In the OG, 20% (n = 1) perceived online boards to be more than 15 min shorter than in-person boards, while 20% (n = 1) of participants in the OG also perceived online boards to be 5 to 15 min longer than in-person boards. In the IG, 20% (n = 2) perceived online boards to be more than 15 min longer than in-person boards. Details can be found in Figure 5. Exploratory testing using Fisher’s Exact or Chi-square tests did not yield significant results (see Table S4 in the Supplementary Material).

A small number of participants provided additional insight through free-text answers into why they perceived there was a difference in duration and/or the participation of junior and senior participants (Table 2).

## 4. Discussion

In summary, this exploratory pilot study provides insight into how prior experience can affect the perceived effectiveness, level of engagement and duration of online tumor boards. These findings reflect perceived effectiveness and not objective performance metrics. While both participants’ groups agreed on several areas, such as the absence of technical barriers and gender parity in involvement, differences in perceptions of communication quality, participant engagement, follow-up management, board duration and format preference emerged. These differences merit further attention and ultimately can provide valuable input for generating hypotheses on the optimization of future medical board meetings.

One key area of difference involved communication challenges. IG participants were more likely to perceive limitations in non-verbal communication and access to digital documentation. This aligns with previous studies emphasizing that while online platforms can facilitate broad accessibility, they often fall short in conveying subtleties like facial expressions and body language, which are essential in collaborative settings [12], and also rely on participants’ willingness to activate their webcam [13]. Studies in remote work settings also underscore the importance of team cohesion for willingness to engage in online formats, indicating a need for prior face-to-face interaction [14,15].

Engagement disparities between junior and senior participants also emerged, with some IG members noting a reduction in perceived involvement for both junior and senior participants in online boards compared to in-person meetings, in contrast to the OG’s experience, who perceived no differences. This observation is concordant with other research, which showed that online meetings and remote work environments can amplify existing power imbalances and create new barriers to equitable participation [16,17]. Our study suggests that, in practice, remote formats may inadvertently stifle participation from certain groups, perhaps due to a more structured and formalized online setup that limits opportunities for spontaneous input. This finding highlights the need for intentional strategies to foster inclusivity and active participation in online boards, particularly for senior members who might be less engaged in virtual settings and for junior participants who may feel less empowered to contribute.

Follow-up management also emerged as an area of divergence: OG participants were more likely to perceive online boards as leading to more cautious and detailed follow-up processes. This finding contrasts with studies suggesting that online environments, while structured, may sometimes lead to reduced follow-up rigor due to decreased accountability in virtual settings [18,19]. However, our preliminary results indicate that for some participants, online boards may indeed encourage a more thoughtful approach to follow-up, potentially because of the recorded, structured nature of online meetings, which enables easier reference to prior discussions.

Beyond follow-up management recommendations, both groups noted uncertainty about the adherence to board decisions. One possible reason for this might be that the patients presented were partly in-house patients and partly outpatients, meaning that not every physician had access to the respective hospital information system.

Duration perceptions in this study were mixed, with most participants in both groups viewing online and in-person boards as similarly timed, though some participants in both groups reported variations in perceived meeting length. This reflects prior research suggesting that virtual meetings often feel shorter or more efficient, likely due to there being fewer informal exchanges [20]. However, the variability in our preliminary results—with some perceiving online boards as both longer and shorter—suggests that individual factors, such as meeting structure, task complexity, and participant engagement, could shape these perceptions. This is consistent with research that showed a higher number of case presentations in online boards [21], which can be underlined by the fact that, after implementation of the online format, our study observed a doubling in the average case load in the board.

Interestingly, while the OG unanimously preferred online boards, the IG showed a more divided preference for board formats, with 30% favoring in-person meetings. This finding may support the literature indicating that preferences for remote or in-person formats are influenced by prior experience and familiarity with each setting [22]. Individuals who experienced both formats might be more aware of the distinct benefits of in-person boards, such as ease of interaction and spontaneous discussion, which are harder to replicate online.

This study has several limitations. Firstly, the small number of participants limits the generalizability of the findings, and the study focuses on perceived and not objective measures. The response rate of 28% introduces potential non-response bias; respondents may be more digitally engaged than those who did not participate. However, the 53 participants who were contacted represented all potential participants who ever attended or expressed interest in attending. From those, only 15 to 20 colleagues consistently attended the boards. This puts the survey participation rate into a much higher bracket of 75% to 100%. Another limitation is the potential for selection bias, as the participants who chose to engage in the study may not represent the broader population, and specialists were more frequently present in the IG, a distribution that is to be expected given the nature of a highly specialized meeting such as pituitary boards. Additionally, the frequent “no opinion” responses and the fact that 53% of participants perceived that they lacked access to information regarding the adherence to board decisions limit the strength of any clinical associations. The anonymity of the participants likely encouraged truthfulness in the reports; however, it also meant that cross-referencing survey responses with clinical roles was not possible. Finally, the temporal gap between the 2020 transition and the 2024 survey may introduce significant recall bias and retrospective perception distortion, potentially influencing the responses provided.

## 5. Conclusions

Overall, our exploratory pilot study contributes new insights to the ongoing discourse on the dynamics of online versus in-person medical boards by detecting nuanced differences in communication, engagement, and preferences that have not been emphasized in the prior literature and provides hypothesis-generating insights. Unlike earlier studies, this study captured distinct perceptions regarding engagement disparities across participant hierarchies and identified a potential link between online formats and more cautious follow-up management. Furthermore, the comparison of groups with differing experiences—OG versus IG—provides a unique lens to understand how familiarity with online-only settings influences perceptions and effectiveness. These exploratory findings provide motivation to conduct larger studies using objective clinical outcome data and formal qualitative coding to validate these initial perceptions, ultimately advancing the field by offering a more granular understanding of the challenges and opportunities in optimizing remote online-only collaboration in medical boards.

## Figures and Tables

**Table 1:** Participant characteristics stratified by group. OG = Online-only group (n = 5); IG = online and in-person group (n = 10). Columns: count (n); within-group percentage (%); exact 95% Clopper–Pearson CI—reported separately for OG, IG, and the full sample (n = 15). Variables: career position (Q1: resident/specialist) and gender (Q2: female/male). Zero-count options are omitted. No inferential test applied; purely descriptive.

Variable	Category	OG n (n = 5)	OG %	OG 95% CI	IG n (n = 10)	IG %	IG 95% CI	Total n (n = 15)	Total %	Total 95% CI
Career Position (Q1)	Resident	4	80	[28.4%, 99.5%]	1	10	[0.3%, 44.5%]	5	33	[11.8%, 61.6%]
Career Position (Q1)	Specialist	1	20	[0.5%, 71.6%]	9	90	[55.5%, 99.7%]	10	67	[38.4%, 88.2%]
Gender (Q2)	Female	4	80	[28.4%, 99.5%]	6	60	[26.2%, 87.8%]	10	67	[38.4%, 88.2%]
Gender (Q2)	Male	1	20	[0.5%, 71.6%]	4	40	[12.2%, 73.8%]	5	33	[11.8%, 61.6%]

**Table 2:** Free-text answers (in brackets: respondent’s group affiliation). Free-text responses were categorized into themes via independent review by two authors to ensure consistency.

If you noted a difference in duration, why do you believe this difference occurred?	less interactive exchange online (OG) more patients discussed (OG) over time, more patient cases were included in the board (IG) less discussion (IG)
If you noted a difference in participation of junior and senior participants, why do you believe this difference occurred?	1. less senior participation online (IG)

## Data Availability

The datasets generated and analyzed during the current study are not publicly available to protect participant privacy but are available from the corresponding author on reasonable request.

## Supplementary Materials

The following supporting information can be downloaded at: https://www.mdpi.com/article/10.3390/jcm15083132/s1, Table S1: Exploratory analysis of technical and communication feasibility of online-only tumor board participation, stratified by group. Table S2: Exploratory analysis of participation dynamics across online tumor board sessions, stratified by group. Table S3: Exploratory analysis of perceived clinical outcomes across online tumor board sessions, stratified by group. Table S4: Exploratory analysis of efficiency and preferences across online tumor board sessions, stratified by group.

## Appendix A

**Table A1:** Questions in both the OG (group that experienced only the online board format) and IG (group that experienced both online and in-person board formats).

What Is Your Current Career Position?	Resident Specialist Other
For How Many Years Have You Been in This Career Position?	
What is your gender?	Female Male Non-binary Prefer not to say
Have you experienced both the online pituitary tumor board format and the former in-person pituitary tumor board format?	Yes No

**Table A2:** Questions in the OG (group that experienced only the online board format).

Were technical issues (e.g., hindrance to access board via link, audio/video problems during board) a frequent hindrance for you to participate in online pituitary tumor boards?	yes no no opinion
If yes, which technical issues did you experience?	
Did you have adequate access to digital documentation and information (e.g., medical history, pathology reports) about the discussed case during online pituitary tumor boards?	yes no no opinion
Do you believe non-verbal cues were adequately conveyed and interpreted during online pituitary tumor boards?	yes no no opinion
Was privacy and confidentiality of patient information adequately maintained during online pituitary tumor boards?	yes no no opinion
Please rate the duration of online pituitary tumor boards compared to other in-person-only boards that you have experienced prior in other settings. Online pituitary tumor boards were…	…about 5–15 min shorter than in-person-only boards …more than 15 min shorter than in-person-only boards …about 5–15 min longer in-person-only boards …more than 15 min longer in-person-only boards …were comparable in duration to in-person-only boards (less than 5 min difference)
If you noted a difference in duration, why do you believe this difference occurred?	
Please rate the level of involvement of junior participants during online pituitary tumor boards compared to other in-person-only boards that you have experienced prior in other settings. During online pituitary tumor boards…	…junior participants were less involved in the decision and implementation process than in in-person only boards …junior participants were more involved in the decision and implementation process than in in-person only boards …involvement of junior participants in the decision and implementation process was the same as in in-person only boards
Please rate the level of involvement of senior participants during online pituitary gland boards compared to other in-person-only boards that you have experienced prior in other settings. During online pituitary gland boards…	…senior participants were less involved in the decision and implementation process than in in-person only boards …senior participants were more involved in the decision and implementation process than in in-person only boards …involvement of senior participants in the decision and implementation process was the same as in in-person only boards
If you noted a difference in participation of junior and senior participants, why do you believe this difference occurred?	
Please rate the level of involvement of female participants during online pituitary tumor boards compared to other in-person-only boards that you have experienced prior in other settings. During online pituitary tumor boards,	…female participants were less involved in the decision and implementation process than in in-person only boards. …female participants were more involved in the decision and implementation process than in in-person only boards. …involvement of female participants in the decision and implementation process was the same as in in-person only boards
Please rate the level of involvement of male participants during online pituitary gland boards compared to other in-person-only boards that you have experienced prior in other settings. During online pituitary gland boards,	…male participants were less involved in the decision and implementation process. …male participants were more involved in the decision and implementation process. …involvement of male participants in the decision and implementation process was the same as in in-person only boards
If you noted a difference in participation of female and male participants, why do you believe this difference occurred?	
Do you believe treatment decisions reached in online pituitary tumor boards were leaning towards a more aggressive approach compared to other in-person-only boards that you have experienced prior in other settings?	yes no no opinion
Do you believe decisions for follow-up management reached in online pituitary tumor boards were leaning towards a more cautious and elaborative follow-up compared to other in-person-only boards that you have experienced prior in other settings?	yes no no opinion
Pituitary tumor board decisions were adhered to…	…more often in online pituitary gland boards …more often in other in-person-only boards that I have experienced prior in other settings …the same in online pituitary gland boards and other in-person-only boards that I have experienced prior in other settings I do not have access to this information
Overall, do you prefer online pituitary gland boards or in-person boards as you experienced them prior in other settings?	I prefer online pituitary gland boards I prefer in-person pituitary gland boards

**Table A3:** Questions in the IG (group that experienced both online and in-person board formats).

Were technical issues (e.g., hindrance to access board via link, audio/video problems during board) a frequent hindrance for you to participate in online pituitary tumor boards?	yes no no opinion
If yes, which technical issues did you experience?	
Did you have adequate access to digital documentation and information (e.g., medical history, pathology reports) about the discussed case during online pituitary tumor boards?	yes no no opinion
Do you believe non-verbal cues were adequately conveyed and interpreted during online pituitary tumor boards?	yes no no opinion
Was privacy and confidentiality of patient information adequately maintained during online pituitary tumor board boards?	yes no no opinion
Please rate the duration of online pituitary gland boards compared to in-person only pituitary gland boards. Online pituitary gland boards were…	…about 5–15 min shorter than in-person-only boards …more than 15 min shorter than in-person-only boards …about 5–15 min longer in-person-only boards …more than 15 min longer in-person-only boards …were comparable in duration to in-person-only boards (less than 5 min difference)
If you noted a difference in duration, why do you believe this difference occurred?	
Please rate the level of involvement of junior participants during online pituitary tumor boards and in-person only pituitary tumor boards. During online pituitary tumor boards…	…junior participants were less involved in the decision and implementation process than in in-person only pituitary gland boards …junior participants were more involved in the decision and implementation process than in in-person only pituitary gland boards …involvement of junior participants in the decision and implementation process was the same as in in-person only pituitary gland boards
Please rate the level of involvement of senior participants during online pituitary tumor boards and in-person only pituitary tumor boards. During online pituitary tumor boards…	…senior participants were less involved in the decision and implementation process than in in-person only pituitary gland boards …senior participants were more involved in the decision and implementation process than in in-person only pituitary gland boards …involvement of senior participants in the decision and implementation process was the same as in in-person only pituitary gland boards
If you noted a difference in participation of junior and senior participants, why do you believe this difference occurred?	
Please rate the level of involvement of female participants during online pituitary tumor boards and in-person only pituitary tumor boards. During online pituitary tumor boards,	…female participants were less involved in the decision and implementation process than in in-person only pituitary gland boards. …female participants were more involved in the decision and implementation process than in in-person only pituitary gland boards. …involvement of female participants in the decision and implementation process was the same as in in-person only pituitary gland boards
Please rate the level of involvement of male participants during online pituitary tumor boards and in-person only pituitary tumor boards. During online pituitary tumor boards,	…male participants were less involved in the decision and implementation process. …male participants were more involved in the decision and implementation process. …involvement of male participants in the decision and implementation process was the same as in in-person only pituitary gland boards
If you noted a difference in participation of female and male participants, why do you believe this difference occurred?	
Do you believe treatment decisions reached in online pituitary tumor boards were leaning towards a more aggressive approach compared to in-person-only pituitary tumor boards?	yes no no opinion
Do you believe decisions for follow-up management reached in online pituitary tumor boards were leaning towards a more cautious and elaborative follow-up compared to in-person-only pituitary tumor boards?	yes no no opinion
Pituitary tumor board decisions were adhered to…	more often following online pituitary gland boards more often following in-person only pituitary gland boards the same following online und in-person only pituitary gland boards I do not have access to this information
Overall, do you prefer online pituitary gland boards or in-person pituitary boards?	I prefer online pituitary gland boards I prefer in-person pituitary gland boards

## References

[1] Jain D.S., Kak D.S. (2024). The Digital Revolution in Healthcare: A Glimpse into the Future. Futuristic Trends in Medical Sciences Volume 3 Book 4.

[2] Lau B., Abreu E., Singh N., Venkataraman T., McParland A., Xin K., Sluka D., Brass J. (2023). A Clinical Psychological Analysis of Virtual Care Adoption During the COVID-19 Pandemic: Digital Resilience in Pain Management. Authorea.

[3] Bradley J.M., Redinger J.W., Tuck M.G., Sweigart J.R., Smeraglio A.C., Mitchell C.A., Laudate J.D., Kwan B.K., Jagannath A.D., Heppe D.B. (2023). A Multicenter Observational Study Comparing Virtual with in-Person Morning Reports during the COVID-19 Pandemic. South. Med. J..

[4] Zhang S., Ma C. (2023). How Has the COVID-19 Pandemic Affected the Utilisation of Online Consultation and Face-to-Face Medical Treatment? An Interrupted Time-Series Study in Beijing, China. BMJ Open.

[5] Bhargava S., Negbenebor N., Sadoughifar R., Ahmad S., Kroumpouzos G. (2021). Global Impact on Dermatology Practice due to the COVID-19 Pandemic. Clin. Dermatol..

[6] Ekhator C., Kesari S., Tadipatri R., Fonkem E., Grewal J. (2022). The Emergence of Virtual Tumor Boards in Neuro-Oncology: Opportunities and Challenges. Cureus.

[7] Johannessen L.E.F. (2024). Anticipated affordances: Understanding early reactions to new technologies. New Media Soc..

[8] Salin L., Koponen J. (2024). Top managers’ media selection and interaction goals in e-leadership. Inf. Technol. People.

[9] Liu C., Messer M., Linardon J., Fuller-Tyszkiewicz M. (2025). Applying models of self-regulated learning to understand engagement with digital health interventions: A narrative review. Front. Digit. Health.

[10] Avila-Garzon C., Bacca-Acosta J. (2023). Predictors of Engagement in Virtual Reality Storytelling Environments about Migration. Appl. Sci..

[11] Cunningham C.T., Quan H., Hemmelgarn B., Noseworthy T., A Beck C., Dixon E., Samuel S., A Ghali W., Sykes L.L., Jetté N. (2015). Exploring physician specialist response rates to web-based surveys. BMC Med. Res. Methodol..

[12] Li Q., Liu Z., Zhang Z., Wang Q., Ma M. (2024). Decoding Group Emotional Dynamics in a Web-Based Collaborative Environment: A Novel Framework Utilizing Multi-Person Facial Expression Recognition. Int. J. Hum. Comput. Interact..

[13] Zabel S., Vinan Navas G.T., Otto S. (2022). Social Norms and Webcam Use in Online Meetings. Front. Psychol..

[14] Turel O., Connelly C.E. (2012). Team Spirit: The Influence of Psychological Collectivism on the Usage of E-Collaboration Tools. Group Decis. Negot..

[15] Maurer M., Bach N., Oertel S. (2022). Forced to Go Virtual. Working-from-Home Arrangements and Their Effect on Team Communication during COVID-19 Lockdown. Ger. J. Hum. Resour. Manag..

[16] Einstein K.L., Glick D., Godinez Puig L., Palmer M. (2023). Still Muted: The Limited Participatory Democracy of Zoom Public Meetings. Urban Aff. Rev..

[17] Yungbluth S.C., Hart Z.P. (2021). The Amplification of Power Dynamics in Virtual Work. Virtual Communities.

[18] Bodger K., Taylor F., Dobson E., Bunn J., Grainger S., Cummings F., Bloom S., Kennedy N. (2022). O28 Factors Associated with Preferences for in-Person versus Remote Consultations for IBD: A UK-Wide Online Survey. Proceedings of the Oral Presentations.

[19] Keating D.J., Cullen-Lester K.L., Meuser J.D. (2024). Virtual Work Conditions Impact Negative Work Behaviors via Ambiguity, Anonymity, and (un)accountability: An Integrative Review. J. Appl. Psychol..

[20] Lacy A., Polsley S., Ray S., Hammond T. (2022). A Seat at the Virtual Table: Emergent Inclusion in Remote Meetings. Proc. ACM Hum. Comput. Interact..

[21] Espín A., Rojas C. (2021). The Impact of the COVID-19 Pandemic on the Use of Remote Meeting Technologies. SSRN Electron. J..

[22] Davis C.H., Ho J., Stephenson R., August D.A., Gee H., Weiner J., Alexander H.R., Pitt H.A., Berger A.C. (2022). Virtual Tumor Board Increases Provider Attendance and Case Presentations. JCO Oncol. Pract..

