# Supplementary material for: Transition from In-Person to Online Boards—An Exploratory Pilot Study on Pituitary Tumor Board Meetings
Source: J Clin Med. 2026 Apr 20;15(8):3132. doi: 10.3390/jcm15083132 (PMC13117692; doi:10.3390/jcm15083132)
Supplement: Supplementary file 1 [file jcm-15-03132-s001.zip › jcm-4179921-supplementary.pdf]

Supplemental Material

Supplementary Tables for Manuscript Title: Transition from in-person to online boards – an exploratory pilot study on pituitary tumor board meetings

Table S1: Exploratory analysis of technical and communication feasibility of online-only tumor board participation, stratified by group. OG = Online Only Group (n=5); IG = Online and In-person Group (n=10). Percentages are within-group. Exact 95% Clopper-Pearson confidence intervals. All statistics are exploratory. P-values calculated using Fisher's Exact Test (2×2) or Chi-square (matrices >2×2).

Q	Category	Survey Item	Res-ponse	Total (n)	Total %	Total 95% CI	OG (n)	OG %	OG 95% CI	IG (n)	IG %	IG 95% CI	Diff (OG-IG)	Cra-mers V	p Value
Q4	Technical Quality	technical issues	no	14	93	[68.1%, 99.8%]	4	80	[28.4%, 99.5%]	10	100	[69.2%, 100.0%]	-20	0.378	0.333
Q4	Technical Quality	technical issues	yes	0	0	[0.0%, 21.8%]	0	0	[0.0%, 52.2%]	0	0	[0.0%, 30.8%]	0	0.000	0.333
Q4	Technical Quality	technical issues	no opinion	1	7	[0.2%, 31.9%]	1	20	[0.5%, 71.6%]	0	0	[0.0%, 30.8%]	20	0.378	0.333
Q3	Technical Quality	access to case digital documentation	no	1	7	[0.2%, 31.9%]	0	0	[0.0%, 52.2%]	1	10	[0.3%, 44.5%]	-10	0.189	0.281
Q3	Technical Quality	access to case digital documentation	yes	13	87	[59.5%, 98.3%]	4	80	[28.4%, 99.5%]	9	90	[55.5%, 99.7%]	-10	0.139	0.281
Q3	Technical Quality	access to case digital documentation	no opinion	1	7	[0.2%, 31.9%]	1	20	[0.5%, 71.6%]	0	0	[0.0%, 30.8%]	20	0.378	0.281
Q6	Communication Quality	non-verbal cues adequately conveyed	no	3	20	[4.3%, 48.1%]	0	0	[0.0%, 52.2%]	3	30	[6.7%, 65.2%]	-30	0.354	0.269
Q6	Communication Quality	non-verbal cues adequately conveyed	yes	8	53	[26.6%, 78.7%]	4	80	[28.4%, 99.5%]	4	40	[12.2%, 73.8%]	40	0.378	0.269
Q6	Communication Quality	non-verbal cues adequately conveyed	no opinion	4	27	[7.8%, 55.1%]	1	20	[0.5%, 71.6%]	3	30	[6.7%, 65.2%]	-10	0.107	0.269
Q5	Communication Quality	privacy maintained	no	1	7	[0.2%, 31.9%]	1	20	[0.5%, 71.6%]	0	0	[0.0%, 30.8%]	20	0.378	0.333
Q5	Communication Quality	privacy maintained	yes	14	93	[68.1%, 99.8%]	4	80	[28.4%, 99.5%]	10	100	[69.2%, 100.0%]	-20	0.378	0.333
Q5	Communication Quality	privacy maintained	no opinion	0	0	[0.0%, 21.8%]	0	0	[0.0%, 52.2%]	0	0	[0.0%, 30.8%]	0	0.000	0.333

Table S2: Exploratory analysis of participation dynamics across online tumor board sessions, stratified by group. OG = Online Only Group (n=5); IG = Online and In-person Group (n=10). Exact 95% Clopper-Pearson CIs are given. Diff: OG% − IG% per response row (positive = higher endorsement in OG). All statistics are exploratory. P-values calculated using Fisher's Exact Test (2×2) or Chi-square (matrices >2×2).

Q	Category	Survey Item	Res-ponse	Total (n)	Total %	Total 95% CI	OG (n)	OG %	OG 95% CI	IG (n)	IG %	IG 95% CI	Diff (OG-IG)	Cra-mers V	p Value
Q7	Participation by Demographics	involvement of male participants	less	0	0	[0.0%, 21.8%]	0	0	[0.0%, 52.2%]	0	0	[0.0%, 30.8%]	0	0.000	N/A
Q7	Participation by Demographics	involvement of male participants	same	15	100	[78.2%, 100.0%]	5	100	[47.8%, 100.0%]	10	100	[69.2%, 100.0%]	0	0.000	N/A
Q7	Participation by Demographics	involvement of male participants	more	0	0	[0.0%, 21.8%]	0	0	[0.0%, 52.2%]	0	0	[0.0%, 30.8%]	0	0.000	N/A
Q10	Participation by Seniority	involvement of junior participants	less	1	7	[0.2%, 31.9%]	0	0	[0.0%, 52.2%]	1	10	[0.3%, 44.5%]	-10	0.189	1.000
Q10	Participation by Seniority	involvement of junior participants	same	14	93	[68.1%, 99.8%]	5	100	[47.8%, 100.0%]	9	90	[55.5%, 99.7%]	10	0.189	1.000
Q10	Participation by Seniority	involvement of junior participants	more	0	0	[0.0%, 21.8%]	0	0	[0.0%, 52.2%]	0	0	[0.0%, 30.8%]	0	0.000	1.000
Q9	Participation by Seniority	involvement of senior participants	less	3	20	[4.3%, 48.1%]	0	0	[0.0%, 52.2%]	3	30	[6.7%, 65.2%]	-30	0.354	0.505
Q9	Participation by Seniority	involvement of senior participants	same	12	80	[51.9%, 95.7%]	5	100	[47.8%, 100.0%]	7	70	[34.8%, 93.3%]	30	0.354	0.505
Q9	Participation by Seniority	involvement of senior participants	more	0	0	[0.0%, 21.8%]	0	0	[0.0%, 52.2%]	0	0	[0.0%, 30.8%]	0	0.000	0.505

Table S3: Exploratory analysis of perceived clinical outcomes across online tumor board sessions, stratified by group. OG = Online Only Group (n=5); IG = Online and In-person Group (n=10). All statistics are exploratory only. Given OG n=5 and IG n=10 (total n=15). P-values calculated using Fisher's Exact Test (2×2) or Chi-square (matrices >2×2).

Q	Category	Survey Item	Response	Total (n)	Total %	Total 95% CI	OG (n)	OG %	OG 95% CI	IG (n)	IG %	IG 95% CI	Diff (OG-IG)	Cra-mers V	p Value
Q13	Clinical Outcomes	more aggressive treatment decisions	no	13	87	[59.5%, 98.3%]	3	60	[14.7%, 94.7%]	10	100	[69.2%, 100.0%]	-40	0.555	0.095
Q13	Clinical Outcomes	more aggressive treatment decisions	yes	0	0	[0.0%, 21.8%]	0	0	[0.0%, 52.2%]	0	0	[0.0%, 30.8%]	0	0.000	0.095
Q13	Clinical Outcomes	more aggressive treatment decisions	no opinion	2	13	[1.7%, 40.5%]	2	40	[5.3%, 85.3%]	0	0	[0.0%, 30.8%]	40	0.555	0.095
Q12	Clinical Outcomes	more elaborative/cautious follow-up decisions	no	11	73	[44.9%, 92.2%]	2	40	[5.3%, 85.3%]	9	90	[55.5%, 99.7%]	-50	0.533	0.098
Q12	Clinical Outcomes	more elaborative/cautious follow-up decisions	yes	3	20	[4.3%, 48.1%]	2	40	[5.3%, 85.3%]	1	10	[0.3%, 44.5%]	30	0.354	0.098
Q12	Clinical Outcomes	more elaborative/cautious follow-up decisions	no opinion	1	7	[0.2%, 31.9%]	1	20	[0.5%, 71.6%]	0	0	[0.0%, 30.8%]	20	0.378	0.098
Q11	Clinical Outcomes	adherence to board decision	no access to this information	8	53	[26.6%, 78.7%]	3	60	[14.7%, 94.7%]	5	50	[18.7%, 81.3%]	10	0.094	0.700
Q11	Clinical Outcomes	adherence to board decision	more in online boards	2	13	[1.7%, 40.5%]	1	20	[0.5%, 71.6%]	1	10	[0.3%, 44.5%]	10	0.139	0.700
Q11	Clinical Outcomes	adherence to board decision	more in in-person boards	0	0	[0.0%, 21.8%]	0	0	[0.0%, 52.2%]	0	0	[0.0%, 30.8%]	0	0.000	0.700
Q11	Clinical Outcomes	adherence to board decision	same	5	33	[11.8%, 61.6%]	1	20	[0.5%, 71.6%]	4	40	[12.2%, 73.8%]	-20	0.200	0.700

Table S4: Exploratory analysis of efficiency and preferences across online tumor board sessions, stratified by group. OG = Online Only Group (n=5); IG = Online and In-person Group (n=10). All statistics are exploratory only. P-values calculated using Fisher's Exact Test (2×2) or Chi-square (matrices >2×2).

Q	Category	Survey Item	Response	Total (n)	Total %	Total 95% CI	OG (n)	OG %	OG 95% CI	IG (n)	IG %	IG 95% CI	Diff (OG-IG)	Cramers V	p Value
Q15	Efficiency & Preferences	duration of online tumor boards	15 minutes shorter	1	7	[0.2%, 31.9%]	1	20	[0.5%, 71.6%]	0	0	[0.0%, 30.8%]	20	0.378	0.263
Q15	Efficiency & Preferences	duration of online tumor boards	5-15 minutes shorter	3	20	[4.3%, 48.1%]	1	20	[0.5%, 71.6%]	2	20	[2.5%, 55.6%]	0	0.000	0.263
Q15	Efficiency & Preferences	duration of online tumor boards	< 5 minutes difference	8	53	[26.6%, 78.7%]	2	40	[5.3%, 85.3%]	6	60	[26.2%, 87.8%]	-20	0.189	0.263
Q15	Efficiency & Preferences	duration of online tumor boards	5-15 minutes longer	1	7	[0.2%, 31.9%]	1	20	[0.5%, 71.6%]	0	0	[0.0%, 30.8%]	20	0.378	0.263
Q15	Efficiency & Preferences	duration of online tumor boards	> 15 minutes longer	2	13	[1.7%, 40.5%]	0	0	[0.0%, 52.2%]	2	20	[2.5%, 55.6%]	-20	0.277	0.263
Q14	Efficiency & Preferences	online format preferred	no	3	20	[4.3%, 48.1%]	0	0	[0.0%, 52.2%]	3	30	[6.7%, 65.2%]	-30	0.354	0.505
Q14	Efficiency & Preferences	online format preferred	yes	12	80	[51.9%, 95.7%]	5	100	[47.8%, 100.0%]	7	70	[34.8%, 93.3%]	30	0.354	0.505
